# Rehabilitation of Edentulous Arch Using All-on-Four Treatment Protocol: A Case Report

**DOI:** 10.7759/cureus.58919

**Published:** 2024-04-24

**Authors:** Grazina Fernandes, Meena Aras, Vidya Chitre, Ivy Coutinho, Kennedy Mascarenhas

**Affiliations:** 1 Department of Prosthodontics and Crown and Bridge, Goa Dental College and Hospital, Panaji, IND

**Keywords:** definitive implant supported prosthesis, interim restoration, immediate loading, implant supported fixed prosthesis, full mouth rebilitation, dental implant, prosthetic rehabilitation, all-on-four

## Abstract

In edentulous arches, alveolar ridge atrophy after tooth extraction is a common problem that affects patient comfort and quality of life. Implant-supported fixed restorations are a well-proven treatment option for edentulism. The concepts of implant dentistry have developed over time to produce better aesthetics and functional results. To reduce cantilever length and enable prostheses with 12 teeth, the all-on-four technique entails inserting two anterior implants axially and distally orienting two posterior implants. Compared to conventional loading, immediate loading offers various benefits without compromising quality. An instantaneous fixed provisional allows patients immediate function and preserves their quality of life while also promoting a high degree of patient satisfaction in terms of aesthetics, phonetics, mastication, and psychological comfort.

## Introduction

The most popular prosthetic therapy for edentulism is a conventional complete denture because it improves speech, masticatory function, comfort, aesthetics, and occlusal support. However, retention, stability, and support are still problems with resorbed ridges [1]. An implant-supported prosthesis is one of the treatment options for replacing a single tooth with full-mouth rehabilitation. Based on the number of implants inserted in completely edentulous persons, the restoration may be fixed or removable. For a patient with complete edentulism or severe periodontal disease, it would be advantageous to restore both function and aesthetics in the same session by immediate loading [2].

Although there are other concepts for full mouth rehabilitation, the all-on-four implant protocol is more popular because of its affordability, anatomical benefits, and higher success rate than other concepts. Malo et al. described it in 2003. It is the concept of placing four implants in the maxillary and mandibular anterior regions of totally edentulous arches to support a provisional, fixed, and immediately loaded full-arch prosthesis. To maximize implant length and avoid anatomic features, the two most anterior implants are positioned axially, while the posterior implants are positioned at an angle [3]. This case report describes the use of the all-on-four concept in the rehabilitation of an edentulous arch to support an immediately loaded fixed prosthesis followed by the fabrication of a definitive prosthesis using digital technology.

## Case presentation

**Figure 1:**
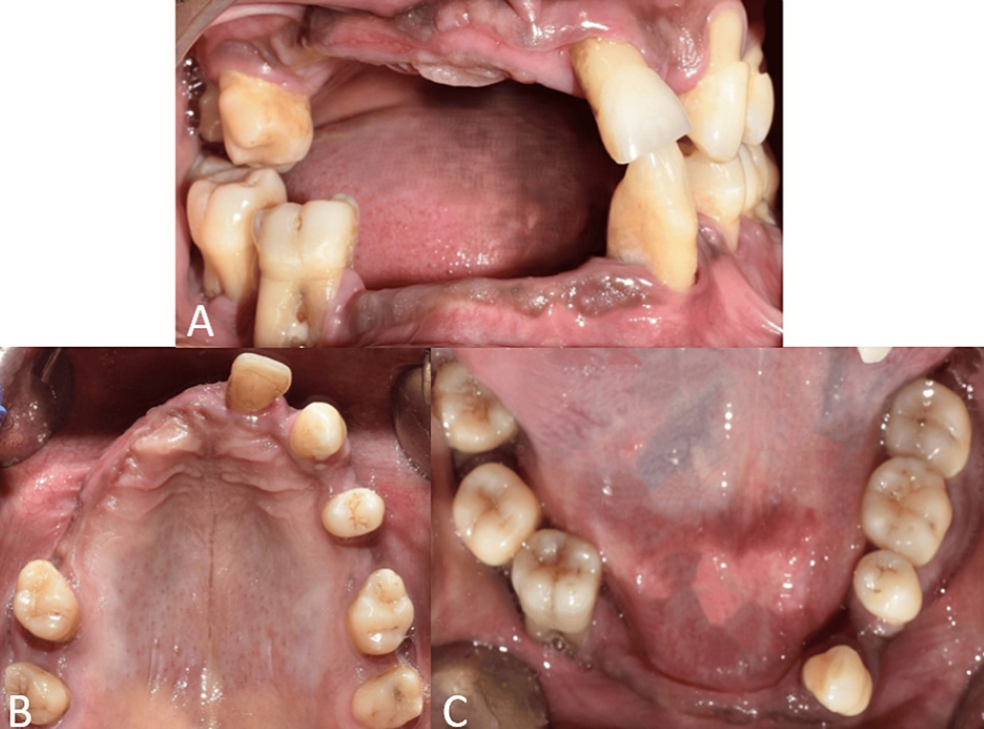
Intraoral view showing severe periodontitis and multiple missing teeth A: Frontal view on maximum intercuspation showing severe periodontitis and multiple missing teeth; B: Occlusal view of maxillary arch showing severe periodontitis and multiple missing teeth; C: Occlusal view of mandibular arch showing severe periodontitis and multiple missing teeth

**Figure 2:**
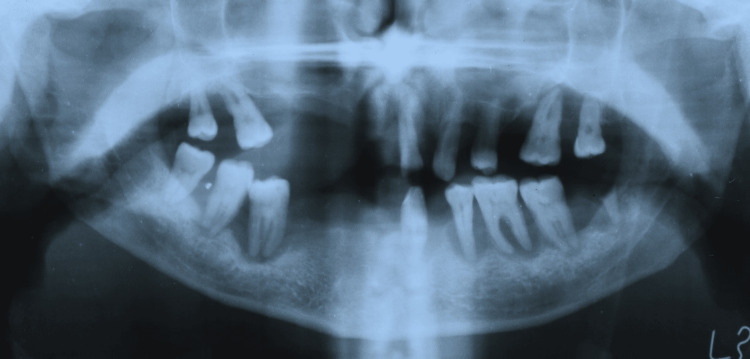
Orthopantomogram showing severe periodontitis and multiple missing teeth

A 42-year-old male patient reported to the Department of Prosthodontics with the chief complaint of inefficient mastication due to multiple mobile and missing teeth. The patient did not have any relevant medical history. An intraoral examination revealed generalized severe periodontitis; missing teeth 11, 12, 13, 14, 15, 16, 22, 24, 26, 31, 32, 34, 38, 41, 42, 43, 44, 45; grade III mobility; and gingival recession of the remaining teeth (Figure 1). The intraoral findings were confirmed with an orthopantomogram (OPG) (Figure 2).

**Figure 3:**
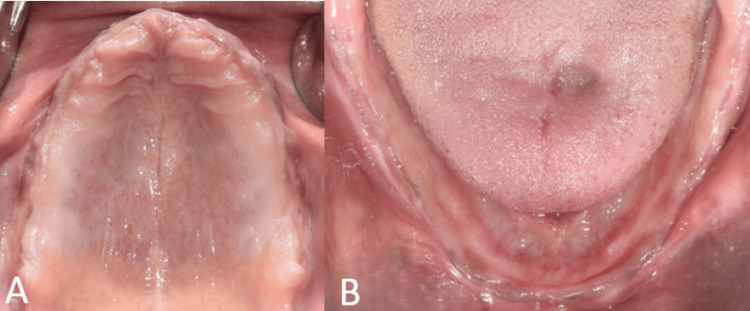
Intraoral view showing edentulous arches A: Maxillary edentulous arch; B: Mandibular edentulous arch

A thorough treatment plan was developed based on clinical and radiological diagnosis. The treatment plan included total extraction of teeth in maxillary and mandibular arches followed by re-evaluation (Figure 3). Several treatment options were discussed with the patient to treat the edentulism. A cone beam computed tomography (CBCT) scan revealed severe resorption of bone. The final treatment plan included a conventional complete denture for the maxilla and an all-on-four protocol for the mandible due to less surgical trauma and cost-effectiveness.

**Figure 4:**
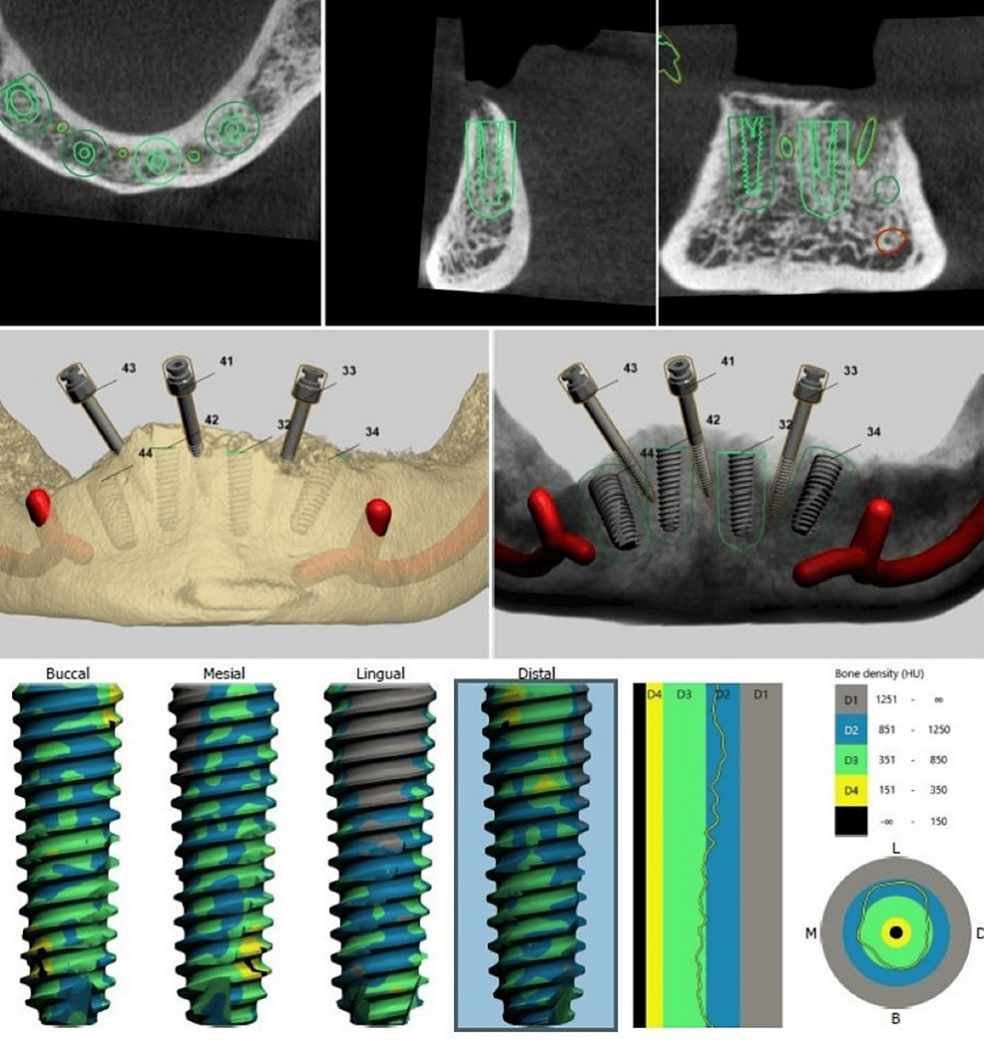
Surgical implant planning

**Figure 5:**
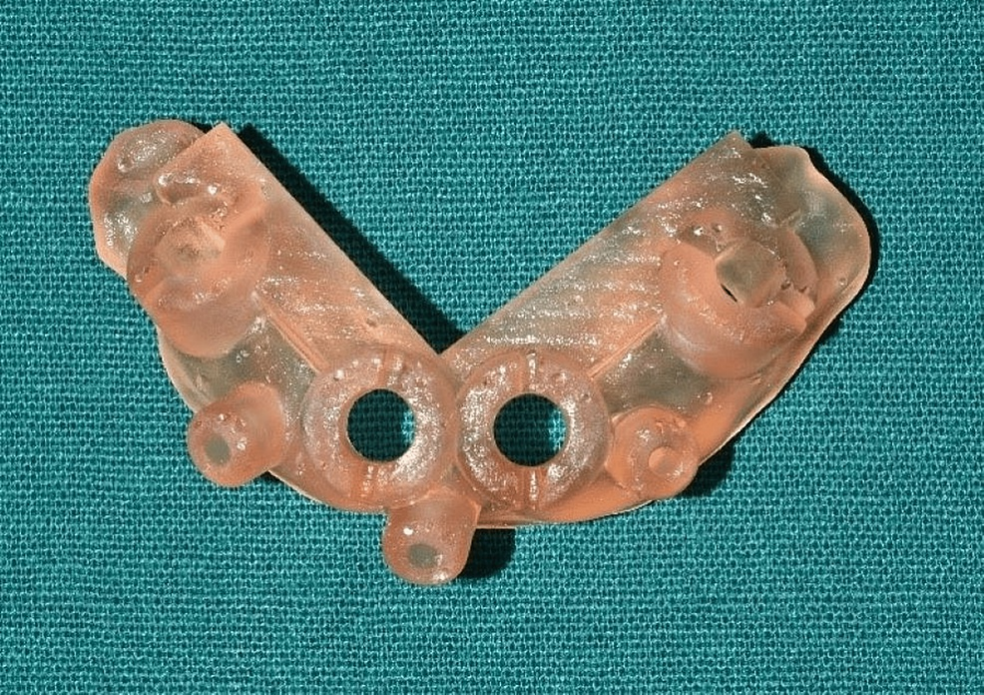
Surgical guide

The presurgical treatment plan included the fabrication of a surgical guide using the 3Shape Dental Software (Copenhagen, DNK) as shown in Figure 4 and Figure 5.

Clinical procedure

Surgical Phase

**Figure 6:**
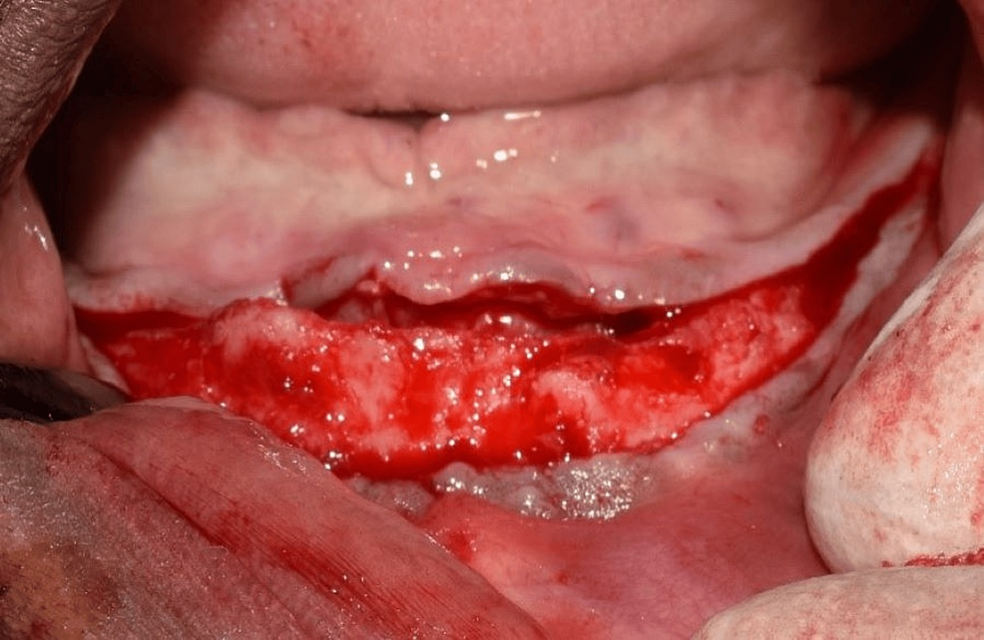
Flap elevation showing the mandibular ridge

Before surgery, the patient was instructed to rinse with 0.2% chlorhexidine for one minute. A 5% povidone-iodine solution was used to disinfect the perioral skin. The surgical procedure was performed under local anesthesia (2% lignocaine with 1:80,000 adrenaline). To gain access, a crestal incision was made from the second mandibular tooth region, continuing to the contralateral side. A full-thickness mucoperiosteal flap was raised (Figure 6). The bone-supported surgical guide was stabilized with anchor pins to the bone and used to direct the placement of the implants. The two 3.5 mm x 11.5 mm (Osstem Implant TS III, Seoul, KOR) anterior implants were positioned perpendicular to the inferior border of the mandible. The two 4.5 mm x 11.5 mm (Osstem Implant TS III) posterior implants were placed distally at a 30° angle. Multi-unit abutments were connected to the implants with 30° angulated abutments for the two posterior tilted implants and straight for the anterior implants. After the placement of the healing caps, the flap was sutured.

Prosthetic Phase for Immediate Loading

**Figure 7:**
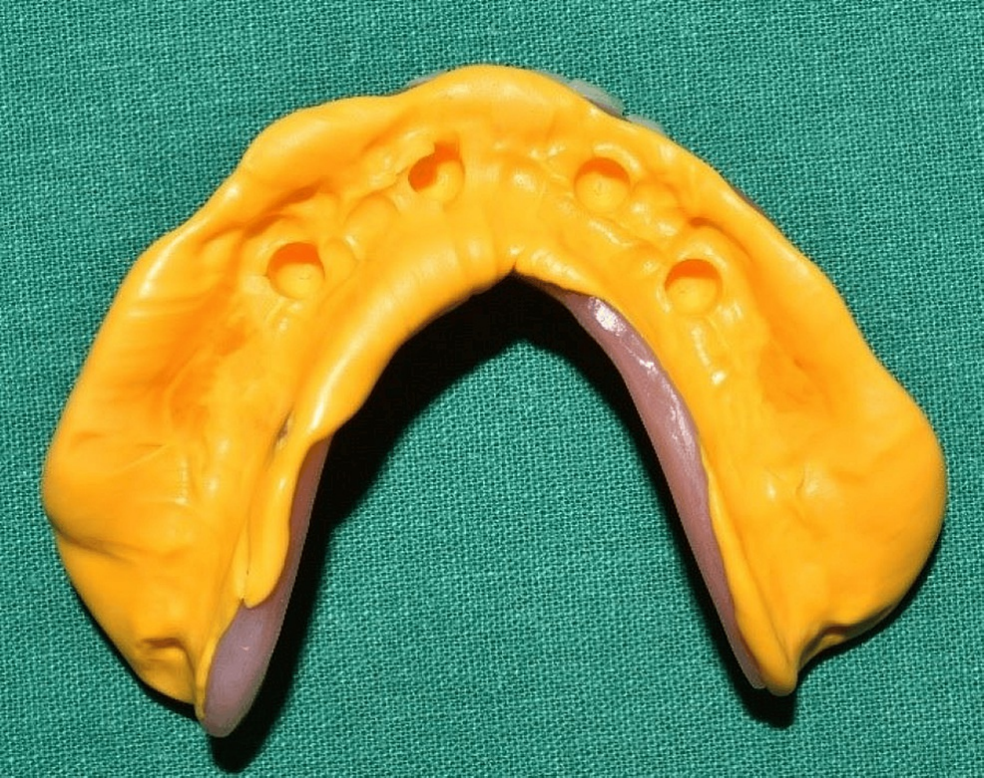
Putty index to locate the healing caps

**Figure 8:**
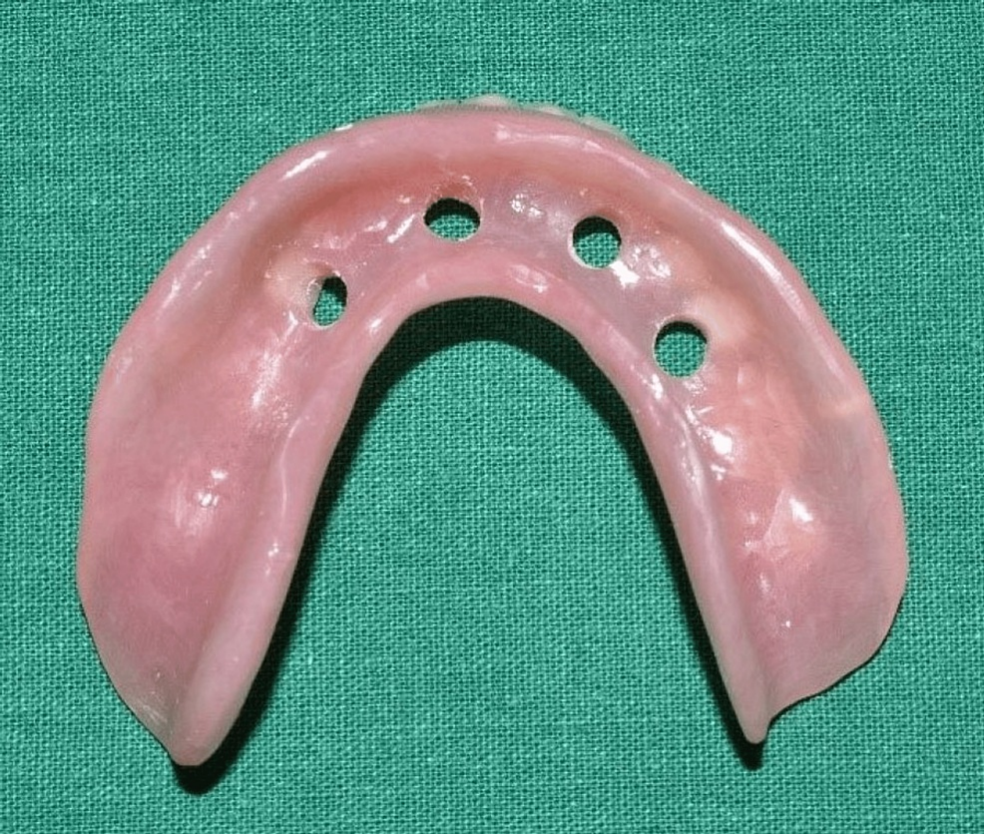
Perforations made in the denture where index markings were present

**Figure 9:**
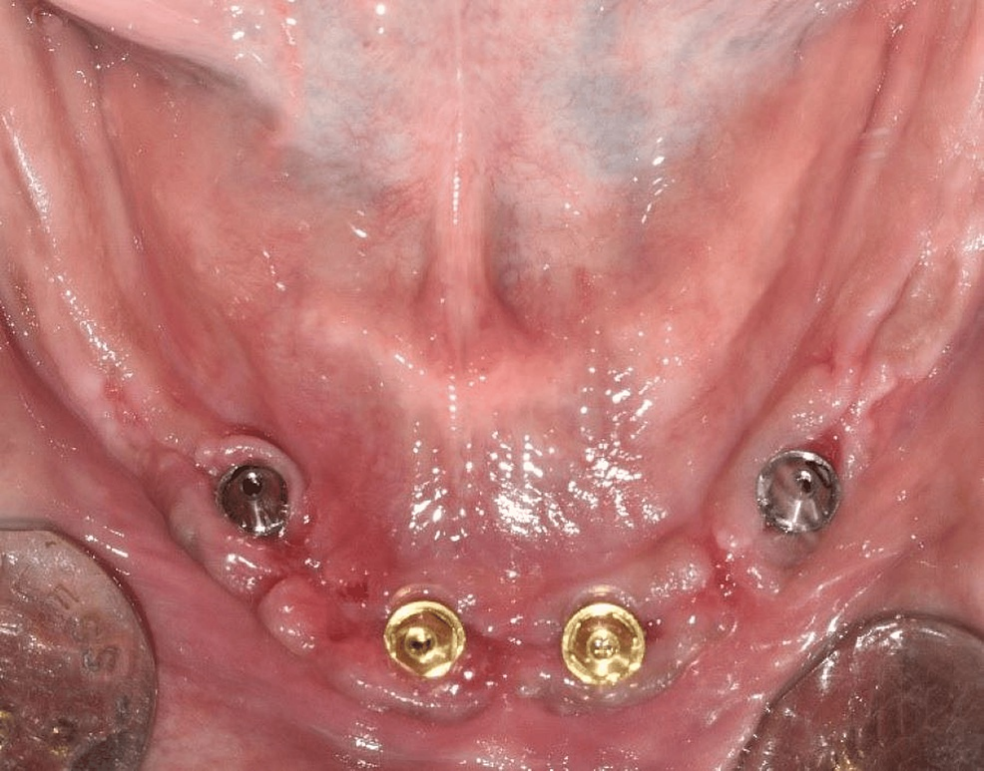
Intraoral view showing multiunit abutments

After 24 hours, a putty impression was made over the healing caps using the prefabricated mandibular complete denture to locate the position of the implants (Figure 7). Thereafter, perforations were made in the denture through the indexed position (Figure 8). The temporary abutments were installed over the multi-unit abutments (Figure 9).

**Figure 10:**
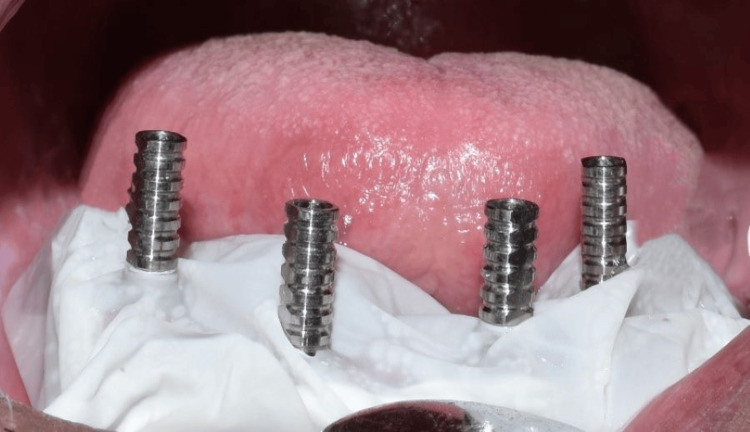
Intraoral view showing placement of a rubber dam over temporary abutments

**Figure 11:**
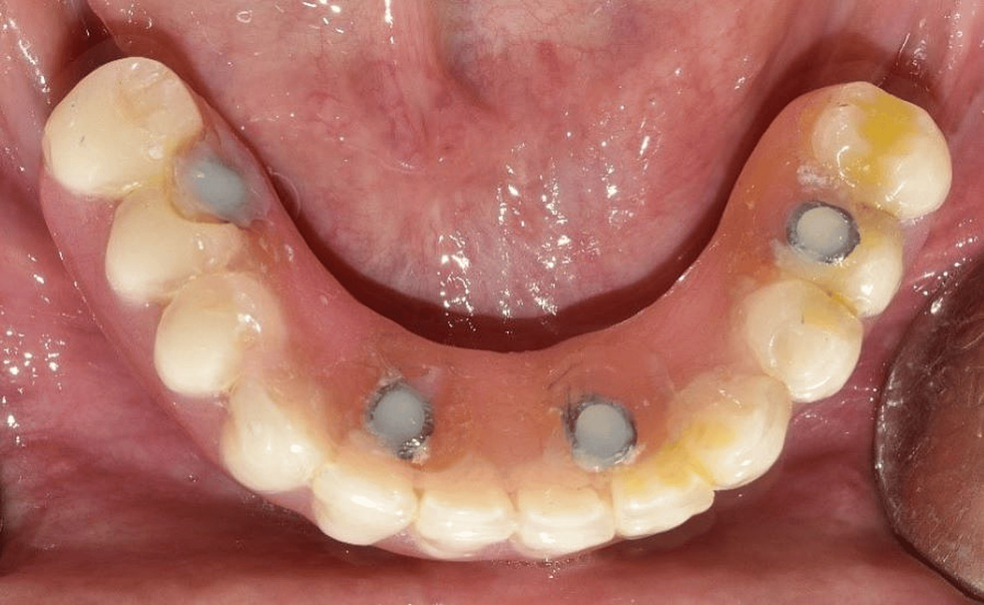
Intraoral view showing immediately loaded provisional prosthesis

A rubber dam was placed over the temporary abutment to protect the mucosa (Figure 10). Following this, an auto-polymerizing resin was added to fix the abutments to the prosthesis. After being polished, the mandibular denture was screwed to the implants to act as an immediate fixed provisional prosthesis (Figure 11).

Prosthetic Phase for Definitive Prosthesis

**Figure 12:**
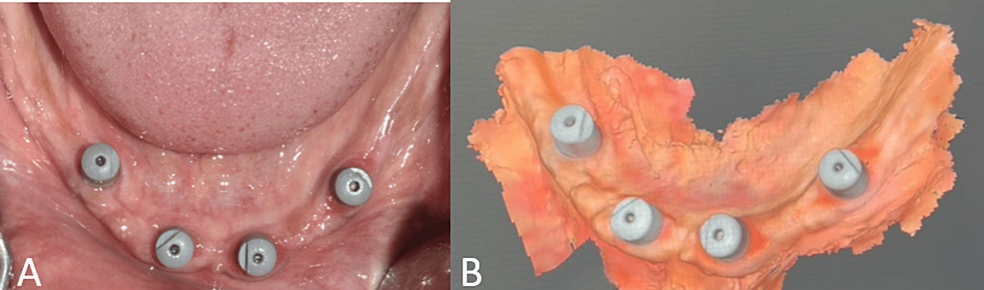
Digital phase A: Intraoral view showing multiunit scan bodies; B: Digital scan showing multiunit scan bodies

**Figure 13:**
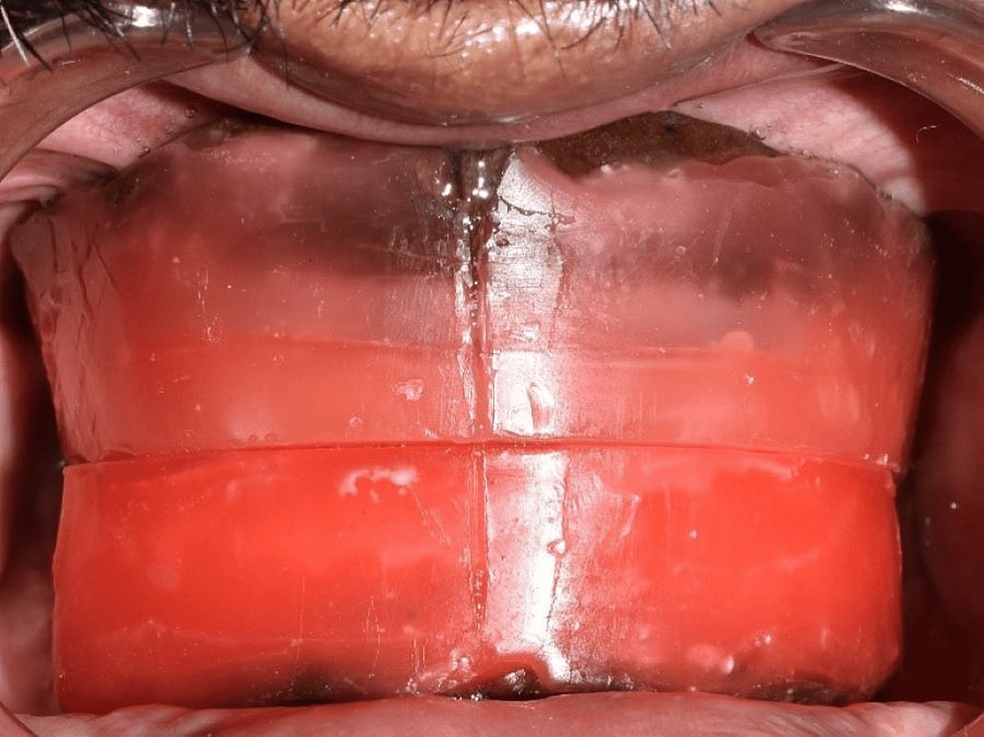
Frontal view showing maxillomandibular jaw relation

**Figure 14:**
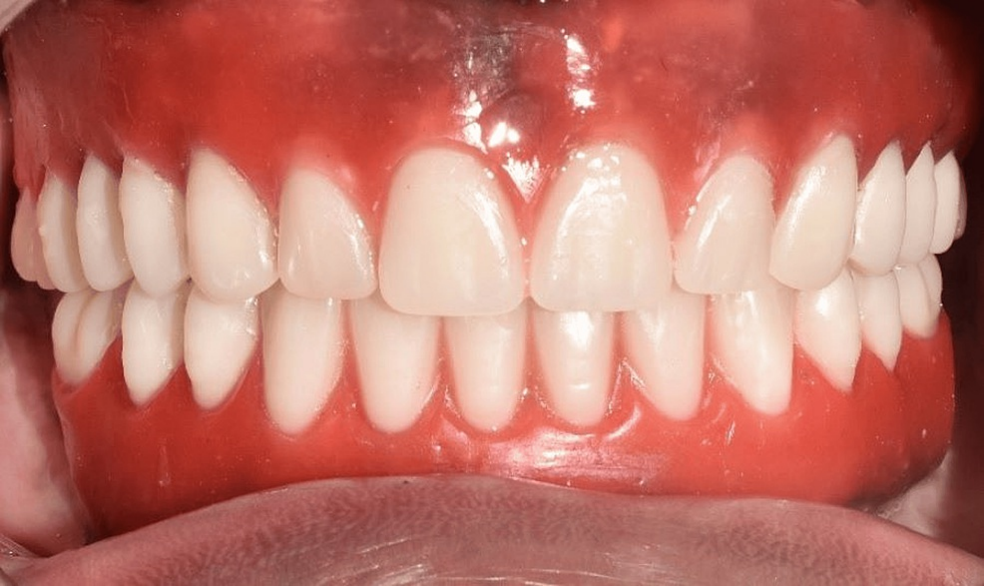
Frontal view showing try-in

The patient was recalled after four months for a definitive prosthesis fabrication. A digital impression was made using multiunit implant scan bodies (Figure 12). The denture bases were fabricated for recording maxillomandibular jaw relation followed by a try-in in the patient's mouth to verify midline, visibility, vertical dimension at rest, and occlusion and freeway space (Figures 13-14).

**Figure 15:**
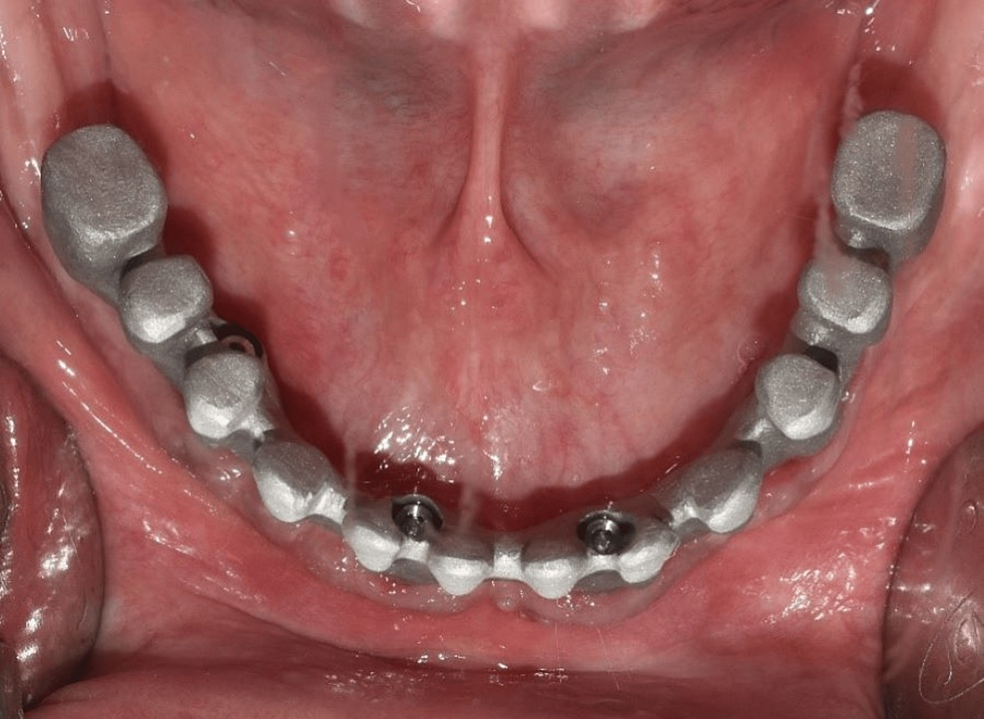
Occlusal view showing metal framework trial

**Figure 16:**
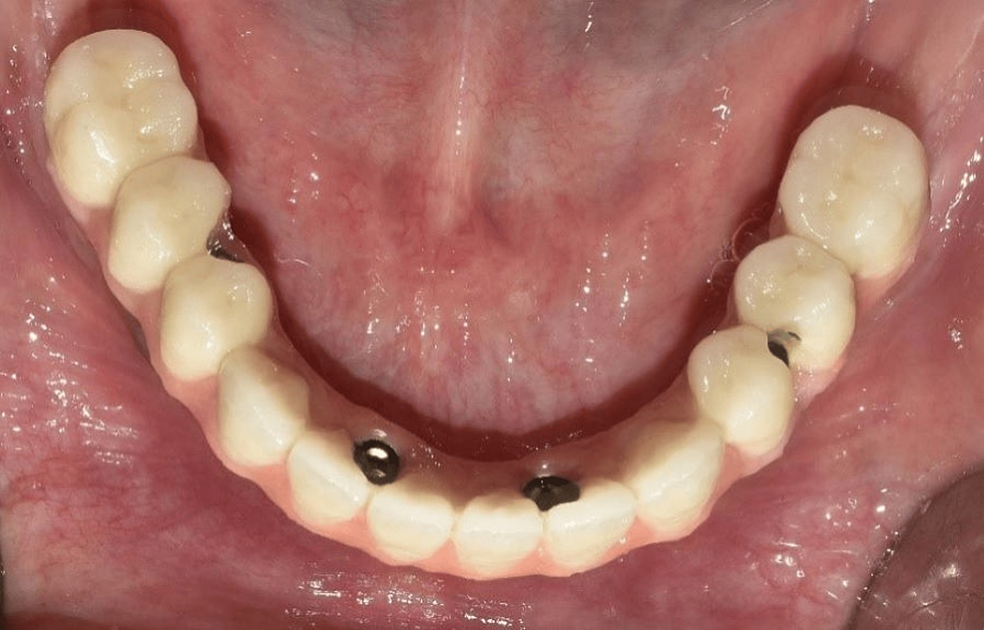
Occlusal view showing framework trial after porcelain veneering

**Figure 17:**
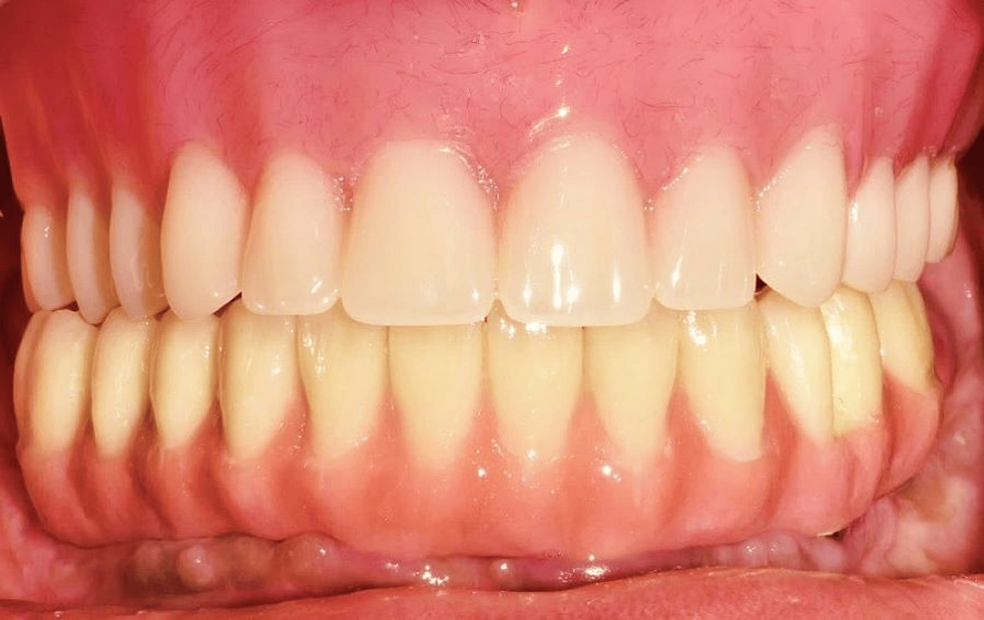
Final implant prosthesis

The cobalt-chromium (Co-Cr) metal framework was fabricated using direct metal laser sintering (DMLS) followed by the intraoral trial (Figure 15) and radiographic evaluation to confirm the passive fit of the framework. Porcelain veneering on the framework with the correct shade as per patient choice was done (Figure 16). The definitive mandibular prosthesis was seated over multiunit abutments and secured using fixation screws (Figure 17). The patient was given oral hygiene instructions.

## Discussion

An atrophic mandible or maxilla is the primary indication of the all-on-four treatment protocol. To insert the four implants, the alveolar process in the maxilla requires minimal dimensions (minimum of 12 mm in height) between the mesial wall of the maxillary sinuses or between the emergence of the mental nerves in the mandible. Another indication is when patients are unwilling to have bone-regenerative treatments such as sinus lift, bone grafting, or dental nerve transpositions, which increase treatment costs and morbidity [4,5]

Dental implants are usually placed vertically; however, completely edentulous arches provide unique difficulties, including limited bone volume, poor quality, and the requirement for bone grafting. By maintaining anatomical characteristics, distal tilting of implants becomes favorable, enabling longer implants with strong cortical anchoring to support the prosthesis. Compared to grafting procedures utilizing conventional axial implants, this method delivers fixed restorations with more biomechanical and therapeutic advantages. The advantages are it can be used in atrophic ridges, immediate loading, less treatment costs and morbidity. Disadvantages include limited bone volume, the anterior-posterior spread being limited by the sinus or the mental foramen, and patients with severe parafunction. Fractures and the loosening of prosthetic components are examples of mechanical complications. Fracture of acrylic prostheses is the most common prosthetic complication. Biological complications include implant failure, peri-implantitis, mucositis, or paresthesia [6,7].

Guided surgery may be more suitable when the implant is to be positioned in close proximity to critical anatomical structures, such as the maxillary sinus, inferior dental nerve, etc. [8]. Guided surgery is the best course of action because of its high degree of precision. While precision attained with guided surgery is better than with freehand surgery, there are limitations as well. These include increased expenses, the need for appropriate anatomical situations for buccal opening, and adequate modification of the surgical guides [9].

Soto et al. found a high survival rate of 99.8% for more than 24 months and a low incidence of complications after the all-on-four protocol [10]. Restoring masticatory function, comfort, and aesthetics is the main goal of surgical techniques such as angled implant insertion or bone regeneration, which will eventually increase social comfort and self-esteem. These objectives are accomplished by implant-supported fixed prostheses that improve success and patient satisfaction levels [11].

## Conclusions

Edentulous patients who have severe ridge resorption and fear complicated surgical procedures can achieve predictable outcomes with the all-on-four treatment protocol. For immediate implant rehabilitation in resorbed ridges to be effective, accurate diagnosis and treatment planning are essential. Immediate loading allows the patient to restore in a very short time both masticatory and phonetic function.
